# Straight but Not Narrow; Within-Gender Variation in the Gender-Specificity of Women’s Sexual Response

**DOI:** 10.1371/journal.pone.0142575

**Published:** 2015-12-02

**Authors:** Meredith L. Chivers, Katrina N. Bouchard, Amanda D. Timmers

**Affiliations:** Department of Psychology, Queen’s University, Kingston, Ontario, Canada; Knox College, UNITED STATES

## Abstract

Gender differences in the specificity of sexual response have been a primary focus in sexual psychophysiology research, however, within-gender variability suggests sexual orientation moderates category-specific responding among women; only heterosexual women show gender-nonspecific genital responses to sexual stimuli depicting men and women. But heterosexually-identified or “straight” women are heterogeneous in their sexual attractions and include women who are exclusively androphilic (sexually attracted to men) and women who are predominantly androphilic with concurrent gynephilia (sexually attracted to women). It is therefore unclear if gender-nonspecific responding is found in both exclusively and predominantly androphilic women. The current studies investigated within-gender variability in the gender-specificity of women’s sexual response. Two samples of women reporting concurrent andro/gynephilia viewed (Study 1, n = 29) or listened (Study 2, n = 30) to erotic stimuli varying by gender of sexual partner depicted while their genital and subjective sexual responses were assessed. Data were combined with larger datasets of predominantly gyne- and androphilic women (total N = 78 for both studies). In both studies, women reporting any degree of gynephilia, including those who self-identified as heterosexual, showed significantly greater genital response to female stimuli, similar to predominantly gynephilic women; gender-nonspecific genital response was observed for exclusively androphilic women only. Subjective sexual arousal patterns were more variable with respect to sexual attractions, likely reflecting stimulus intensity effects. Heterosexually-identified women are therefore not a homogenous group with respect to sexual responses to gender cues. Implications for within-gender variation in women’s sexual orientation and sexual responses are discussed.

## Introduction

Over a decade ago, research reporting on gendered specificity of sexual arousal proposed that women’s and men’s sexual response patterns were fundamentally different [1]. Whereas men’s responses were gender-specific, women—lesbian and heterosexual—showed significant genital response to stimuli depicting both their preferred and nonpreferred genders, a pattern described as *gender-nonspecific*. Since that time, this gender difference in sexual response patterns has been shown to be robust, demonstrated across a range of studies using genital and subjective measures of sexual response to gender cues (e.g., [2–10]). Among studies using other methods to assess sexual interests and processing of sexual cues, this pattern has been replicated such that men show greater gender-specificity in visual attention [11–14], choice reaction time [15, 16], pupil dilation [17, 18], viewing time [19–22], magnetoencephalographic assessment of visual evoked magnetic fields [23], electroencephalographic assessment of contingent negative variation [24] and early cortical processing of nude body stimuli [25, 26], and fMRI assessment of limbic and visual processing regions ([27]; but see also Ponseti et al. [28] for gender-specific neural responses to decontextualized stimuli).

Subsequent research examining sexual orientation effects on the gender-specificity of sexual responses has revealed an even more intriguing puzzle: Only heterosexual women show gender-nonspecific patterns of genital response; gynephilic women, like gay or heterosexual men, show gender-specific sexual responses [4]. The increasing inclusion of women with diverse sexual attractions in sexual psychophysiology research has revealed similar patterns. Other studies also suggest that non-heterosexual women have gender-specific responses across multiple methodologies such as choice reaction time [15, 16], viewing time [19, 21, 22, 29], pupil dilation [18], and early cortical processing of nude body stimuli [26]; all of these studies report gender-specific responses among lesbian women (or “slight to moderately homosexual” women in Hietanen and Nummenmaa [26])–greater response to female versus male stimuli—as compared to gender-nonspecific responses among heterosexual women.

Collectively, this body of research on the gender-specificity of sexual interests, sexual attractions, and sexual arousal suggests that gender—whether participants identify as women or men—is not the most salient moderator of the relationship between sexual attractions and sexual responses. Instead, within-gender variation in sexual attractions and sexual identities is more strongly associated with the specificity of sexual response among women. Limitations of these previous studies, however, obscure the relationship between sexual attractions and sexual response. For example, sexual orientation (i.e., gynephilia, or sexual attraction to women, and androphilia, or sexual attraction to men), and sexual identity (e.g., self-description as heterosexual, bisexual, lesbian, etc.) are typically conflated, with sexual orientation and sexual identity used interchangeably to characterise women’s sexual attractions. This practice is inadvisable given the accumulating evidence of the fluidity of women’s sexuality in terms of congruence between sexual attractions and sexual identities (see Chivers [30, 31]; [32]), such that sexual identities are not reliable indicators of directions of gender attractions among women. Moreover, women reporting both androphilia and gynephilia—bisexual or andro/gynephilic women hereafter—are typically excluded (e.g., [16]), or grouped with predominantly heterosexual/androphilic or lesbian/gynephilic women in analyses [4]. In other studies, sexual orientation is operationalized as tripartite sexual attractions or sexual identities—heterosexual/bisexual/lesbian—versus a continuum of gender attractions (e.g., [21, 22]). Although variability in how the constructs of sexual attractions, identities, and orientations are operationalized often reflects the challenges associated with recruiting sufficiently large samples of sexually diverse women, convenience sampling and classification decisions of this nature may produce a biased picture of women’s sexual responses.

Recently, among public health (e.g., [33]) and sexual orientation scholars (e.g., [34, 35]), the most prevalent sexual minority has been identified as “mostly heterosexual” women, that is, women who report predominant androphilia with some concurrent gynephilia (20% of women in Vrangalova & Savin-Williams [36]); concurrent andro/gynephilic attractions are much more common among women than exclusive gynephilic attractions [36–38]. Instead of adopting the sexual identity “heterosexual”, women with predominant androphilia self-identify as “mostly heterosexual”, or “straight, but not narrow”, and are distinct from other women in terms of their sexual and mental health profiles and their developmental trajectories [35]. Savin-Williams and Vrangalova [35] proposed that pooling data from “mostly heterosexual women” with exclusively heterosexual/androphilic women may obscure important group differences salient to understanding factors associated with sexual attractions.

Only one previous study has examined sexual responses as a function of exclusive versus predominant androphilia in heterosexual women. Suschinsky, Lalumière, and Chivers [9] reported comparisons between 15 predominantly and 5 exclusively androphilic women on gender-specificity indices for genital and subjective sexual arousal. Gender-specificity indices were calculated by subtracting responses to male-male coupled sex from responses to female-female coupled sex, therefore positive indices indicated greater sexual response to female stimuli and negative indices greater sexual response to male stimuli. No significant group differences in gender-specificity were demonstrated for genital response or subjective sexual arousal. Using a stimulus-specificity index (peak sexual arousal to any sexual category minus the sum of sexual arousal to all other sexual categories), predominantly androphilic women showed nonsignificantly greater specificity in their genital responses (*d* = .85), but significantly less specificity in subjective sexual arousal (*d* = 1.63) than exclusively androphilic women. Across all women, approximately half showed greatest genital response to male sexual stimuli. These findings suggest potentially meaningful differences in the sexual response patterns of exclusively and predominantly androphilic women.

The present studies were conducted to examine within-gender variation in the gender-specificity of women’s genital and self-reported sexual responses. We were particularly interested in examining response patterns across the spectrum of gendered attractions, with a focus on gender-specificity of sexual responses among exclusively versus predominantly androphilic women. If gender-nonspecific sexual response is characteristic of heterosexually-identified women, such that within-gender variation in sexual response is associated with sexual identity, we would expect no differences in the sexual response patterns of exclusively versus predominantly androphilic women because they both typically self-identify as heterosexual. Alternatively, if gender-nonspecific response is associated with sexual attractions, that is, degrees of andro- and gynephilia, we would expect exclusively versus predominantly androphilic women to show differing patterns of sexual response. Indeed, the gender-nonspecific sexual responses consistently reported in the literature might reflect the heterogeneity of sexual attractions among heterosexually-identified women, such that exclusively androphilic women would demonstrate gender-specific responses, whereas predominantly androphilic women would show gender-nonspecific responses.

In the first study, women with andro/gynephilic attractions viewed erotic films varying by the gender of the actors (female, male) and sexual activities depicted (nude exercise, masturbation, coupled sex) while their genital responses and subjective sexual arousal were assessed. To examine patterns of sexual response as a function of sexual attractions, that is, andro- and gynephilia, we combined these data with those from a previous sample [4] to create groups of women reporting exclusive androphilia, predominant androphilia (with some concurrent gynephilia), andro/gynephilia (bisexual attractions or significant degrees of both gyne- and androphilia), and predominant/exclusive gynephilia. In the second study, a new sample of women with andro- and gynephilic attractions listened to audiotaped narratives describing sexual encounters with women and men in varying relationship contexts (strangers, friends, long-term romantic partners) while their sexual responses were assessed. Similar to Study 1, we combined these data with those from a previous sample of exclusively and predominantly androphilic women [5], and created groups of women as in Study 1 to examine patterns of sexual response to less intense, nonvisual stimuli (narratives describing sexual activities with women and men) as a function of sexual attractions. For both audiovisual and narrative sexual stimuli, we predicted that sexual responses would vary with degrees of andro- and gynephilia such that exclusively androphilic women would demonstrate gender-specific sexual responses, both genital and subjective, and predominantly androphilic women would show gender-nonspecific sexual responses, as would andro/gynephilic women. Following literature showing gender-specific patterns of sexual response among gynephilic women, we expected the same to emerge from the current data sets.

## Study 1

### Materials and Methods

Ethics approval for this research was granted by the Health Science Research Ethics Board at Queen’s University, and the University of Toronto Health Science Network Research Ethics Board. Participants gave written informed consent after orientation to the laboratory setting, study apparati, and study procedures, and after any questions were addressed.

#### Participants

Twenty-nine cisgender women were recruited through advertisements posted on Queen’s university campus. Inclusion criteria were: between the ages of 18 years and 50 years; able to read and write English fluently; no history of or current psychiatric disorder or substance abuse; no current use of medications known or suspected to influence sexual functioning (see Meston and Frolich [39]); no active sexually transmitted infection; not pregnant; regular menstrual cycles; no sexual response difficulties; had experienced vaginal penetration during sexual activity, tampon use, or a pelvic examination; and reported concurrent andro- and gynephilia.

Data were pooled with those of Chivers et al. [4] for a total *N* of 78; demographic information is based on 76 women, corresponding with usable subjective sexual arousal data. Women ranged in age from 18 to 39 years, with a mean age of 23.79 years old (*SD* = 5.59). The majority of participants (65.8%) were single, 18.4% were in dating relationships, 9.2% were common law or married, and 5.3% were separated or divorced. One participant did not provide her relationship status (1.3%). The plurality of the sample (34.2%) stated that they were of Asian ethnicity, 25.0% of women identified as European, 15.8% as African, 10.5% as Pacific Islander, 5.3% as First Nations, and 1.3% as Hispanic. The remaining 6.6% of women reported a mixed ethnicity, or identified with a heritage not already mentioned above. One participant did not provide her ethnicity (1.3%). The majority of the sample had attended or completed post-secondary education; participants were completing or had completed a bachelor’s degree (78.9%), a graduate or professional degree (5.3%), or community college (10.5%). The remaining participants had completed vocational, trade, or business school (1.3%) or graduated from high school or equivalent (4.0%). All participants received $25 for their time and expenses.

*Sexual Attractions and Sexual Identity*. Participants reported relative sexual attractions to men and women using a variation of the Kinsey Sexual Attraction Scale [40] and were grouped based on their degree of andro/gynephilic attractions: exclusive androphilia (Kinsey 0; *n* = 1), predominant androphilia, with some concurrent gynephilia (Kinsey 1; *n* = 7), andro/gynephilia (Kinsey 2–4; *n* = 15), and predominant/exclusive gynephilia (Kinsey 5–6; *n* = 6). Participants from Chivers et al. [4] were comprised of women reporting exclusive androphilia (Kinsey 0; *n* = 13), predominant androphilia (Kinsey 1; *n* = 12), andro/gynephilia (Kinsey 2–4; *n* = 6), and predominant/exclusive gynephilia (Kinsey 5–6; *n* = 16). For further demographic information, see Chivers et al. [4]. Women endorsed one or more of the following sexual identities; heterosexual, lesbian, bisexual, queer, other, no label. See Table 1 for a breakdown of sexual identities by sexual attraction groups.

**Table 1:** Sexual identities by attraction group for Study 1.

Attraction Group	Sexual Identity					
	Heterosexual	Bisexual	Lesbian/Gay	No Label	Other	Total *n*
Exclusive Androphilia	14	0	0	0	0	14
Predominant Androphilia	14	1	0	2	2*	19
Andro/Gynephilia	2	7	1	5	6**	21
Predominant/Exclusive Gynephilia	0	2	14	1	4***	22

*sexual; heteroflexible.

** bicurious; queer; queer; omnisexual; bisexual but will not marry in the future; pansexual.

*** queer; queer; queer; wouldn’t identify myself as anything.

#### Apparatus and Materials

*Data acquisition*. All psychophysiological responses were sampled and recorded with a Limestone Technologies Data- Pac_USB system (Limestone Technologies, Kingston, Ontario, Canada). The Limestone software and hardware were installed on a Pentium Dell desktop computer (Dell Canada Inc., North York, Ontario, Canada).

*Genital responses*. Women’s genital responses were assessed using vaginal photoplethysmography [41]. The alternating current component of the vaginal photoplethysmograph signal, vaginal pulse amplitude (VPA), was selected as the dependent measure for this study. VPA represents the phasic changes in vaginal blood flow associated with each heartbeat, such that higher amplitudes reflect greater vaginal vasocongestion. Increases in VPA are specific to sexual response [9, 42]. The photoplethysmograph signal was sampled at a rate of 10 samples per second, band-pass filtered (0.5 Hz to 10 Hz), and digitized (40 Hz). VPA was measured as peak-to-trough amplitude for each vaginal pulse. Movement artifacts were detected by visual inspection of the waveforms and removed prior to further data preparation and analysis.

*Subjective sexual arousal*. Immediately before and after each experimental stimulus, women answer the question “*How sexually aroused do you feel*?” using a 10-point Likert-type scale, ranging from 0 (e.g., “no arousal at all”) to 9 (e.g., “most arousal ever experienced/arousal associated with orgasm”). Participants entered their responses using a keypad attached to the armrest of a comfortable reclining chair.

*Experimental stimuli*. The stimuli were those used by Chivers et al. [4], and consisted of 16, 90 second film clips presented with sound, representing eight stimulus categories: control (landscapes accompanied by relaxing music), female nonsexual activity (nude exercise), female masturbation, female–female coupled sex (cunnilingus and vaginal penetration with a strap-on dildo), male nonsexual activity (nude exercise), male masturbation, male–male coupled sex (fellatio and anal intercourse), and female–male coupled sex (cunnilingus and penile-vaginal intercourse). Participants saw two exemplars of each stimulus category. All stimuli were excerpted from commercially available films. A 3-min film with nonsexual content (depictions of landscapes and buildings) was used as an adaptation stimulus.

*Procedure*. Procedures were identical to those described in Chivers et al. [4]. Potential participants responded to advertisements, were screened for eligibility, and scheduled to attend testing sessions that were predominantly held in the late afternoon or early evening. All participants were asked to refrain from sexual activity of all types for 24 hours, physical exercise of all types for one hour (exercise produces sympathetic nervous system arousal that can potentiate genital responses [39]) and asked to refrain from using alcohol or other recreational drugs on the day of testing. Participants confirmed compliance with these requests. Participants gave written informed consent after orientation to the laboratory setting, study apparati, and study procedures. Prior to testing, the experimenter instructed the participant on how to insert the vaginal photoplethysmograph, and how to register subjective responses using the keypad. She also asked the participant to pay full attention to the audio clips, to not touch their genitals or manipulate their genital responding in any way, and to sit as still as possible, to reduce movement artifacts in VPA data [43].

The participants, seated in a comfortable reclining chair in a dimly lit room, inserted the vaginal photoplethsymograph themselves, in private, and watched a 3 minute-long adaptation stimulus presented on a computer monitor approximately 1.5m from the chair. Participants then viewed the experimental stimuli in a predetermined, random order. Immediately before and after every stimulus, participants evaluated and reported on their subjective sexual arousal and affective state. During an inter-stimulus period of approximately three minutes, participants were instructed to relax, to allow their genital arousal to return to its pre-trial baseline. If genital response did not return to pre-trial levels, the participant was asked to engage in a distraction task (e.g., reading aloud from a neutral magazine, counting backwards by 7 from 300) for up to three minutes or until genital responding returned to baseline levels. After the sexual arousal assessment was completed, participants completed the questionnaires and measures described above. Genital gauges underwent high-level disinfection between uses [44].

#### Data Exclusion and Reduction

Subjective sexual arousal was assessed using a change score, subtracting prestimulus from poststimulus ratings of sexual arousal; this method was chosen because change scores are less prone to impression management biases (see Huberman et al. [45]) than post-stimulus measures. Change in genital response was calculated by subtracting pretrial baseline genital response—established during the 5–10 s interval recorded while the participant was completing pretrial questions—from mean genital response to each experimental stimulus. The resulting change scores were standardized within subjects (i.e., ipsatized) to control for individual variability in responding [46]. Z-scores were derived using genital responses from the entire set of 16 experimental stimuli, including the neutral and female-male coupled sex stimuli. Archival genital data from two women were excluded because of equipment problems or unclear VPA signals. The remaining women (*n* = 76) demonstrated a 0.5 SD or greater increase in their maximum genital response to any sexual stimulus and were therefore included in the genital response data analyses [1, 4, 5]. Mean genital and subjective sexual arousal were calculated for each stimulus category by averaging across both category exemplars, and genital and subjective sexual response to gender cues was assessed by averaging across sexual activities (nude exercise, masturbation, coupled sex) for female and male stimuli. For coupled sex stimuli, female-female intercourse was included as a female sexual stimulus and male-male intercourse as a male sexual stimulus; both exemplars depicting female-male coupled sex excluded from the analyses. Preliminary analyses for both genital and subjective sexual responses were conducted prior to collapsing Stimulus Gender across Sexual Activity to check for three-way interactions between Sexual Activity, Stimulus Gender, and Attraction Group, and none were detected. Data analyses were performed using SPSS (SPSS Inc., Chicago, Il, USA; Version 19.0).

### Results

#### Subjective Sexual Arousal

Mean subjective arousal was submitted to a 2 (Stimulus Gender: female, male) X 4 (Attraction Group: exclusive androphilia, predominant androphilia, andro/gynephilia, predominant/exclusive gynephilia) mixed-model ANOVA, revealing a significant main effect of Stimulus Gender, *F*(1, 72) = 51.36, *p* < .001, *η*_*p*_^*2*^ = .42 and a significant interaction between Stimulus Gender and Attraction Group, Wilks’ *Λ* = .793, *F*(3, 72) = 6.26, *p* = .001, *η*_*p*_^*2*^ = .207. There was no significant main effect of Attraction Group, *F*(3, 72) = 1.10, *p* = .36, *η*_*p*_^*2*^ = .044.

**Fig 1:**
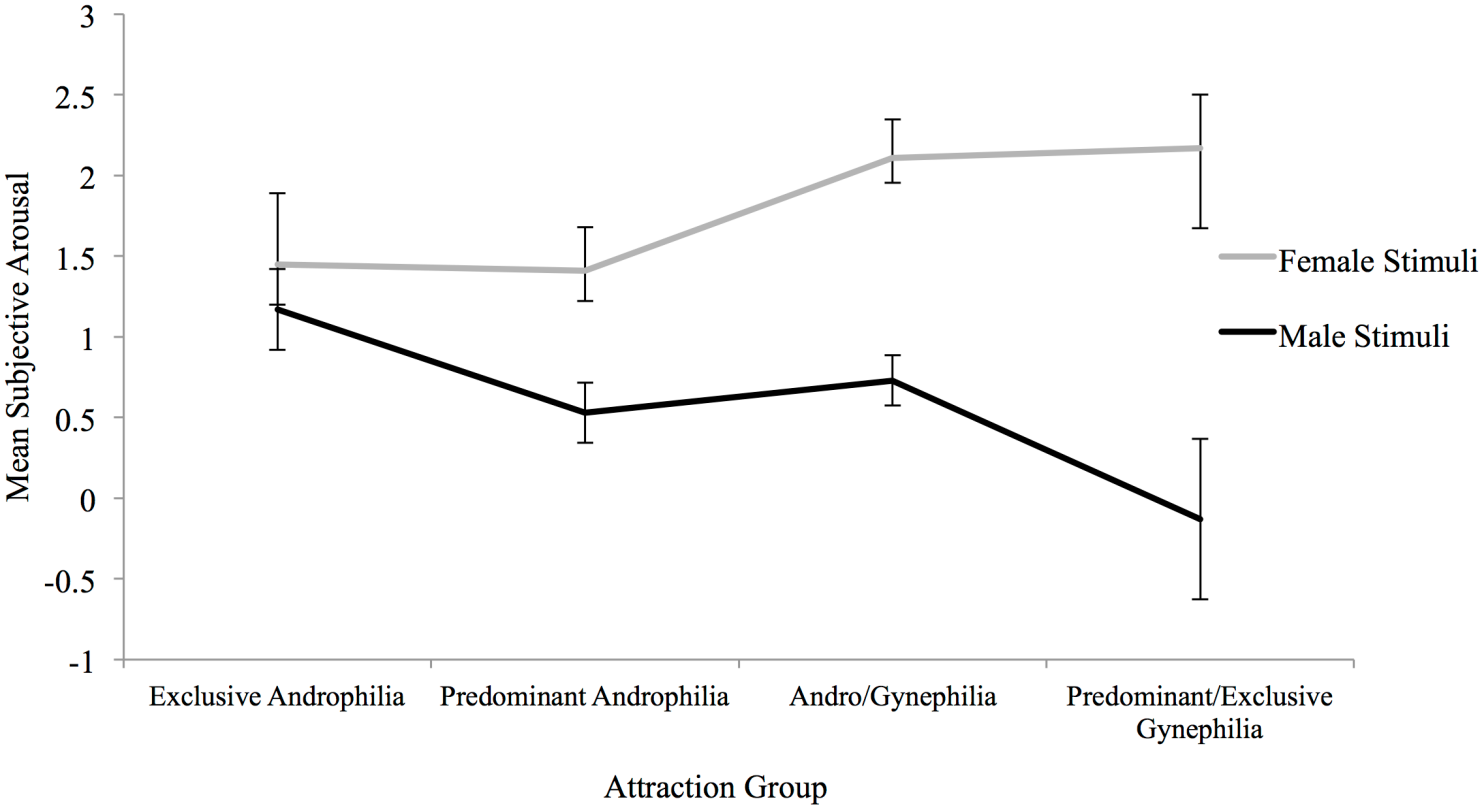
Subjective sexual arousal to female and male audiovisual stimuli by attraction group for Study 1. Exclusive Androphilia (*n* = 14), Predominant Androphilia (*n* = 19), Andro/Gynephilia (*n* = 21), Predominant/Exclusive Gynephilia (*n* = 22). Error bars indicate standard error of the mean.

Mixed-model *t* tests were conducted to follow-up the significant interaction, evaluating the four pairwise differences among the female and male means for subjective sexual arousal to Stimulus Gender with respect to Attraction Group. Predominantly androphilic women, andro/gynephilic women and predominantly/exclusively gynephilic women reported significantly greater subjective sexual arousal to female than to male stimuli, whereas exclusively androphilic women reported gender-nonspecific subjective sexual arousal to male and female stimuli (Fig 1). Effect sizes increased with degree of gynephilia (Table 2). Bivariate linear regressions showed that Kinsey sexual attraction ratings significantly predicted subjective sexual arousal to female, *B* = .17, *t*(74) = 2.55, *p* = .01, and male sexual stimuli, *B* = -.22, *t*(74) = -3.44, *p* = .001, such that gynephilic attractions significantly predicted increases in subjective sexual arousal to female stimuli and decreases in arousal to male stimuli.

**Table 2:** Subjective sexual arousal to gender cues by attraction group for Study 1.

Attraction Group	*n*	*t*	*p*	*d*
Exclusive Androphilia	14	.63	.53	.19
Predominant Androphilia	19	2.30	.02	.87
Andro/Gynephilia	21	3.78	< .001	1.47
Predominant/Exclusive Gynephilia	22	6.46	< .001	1.72

#### Genital Response

**Fig 2:**
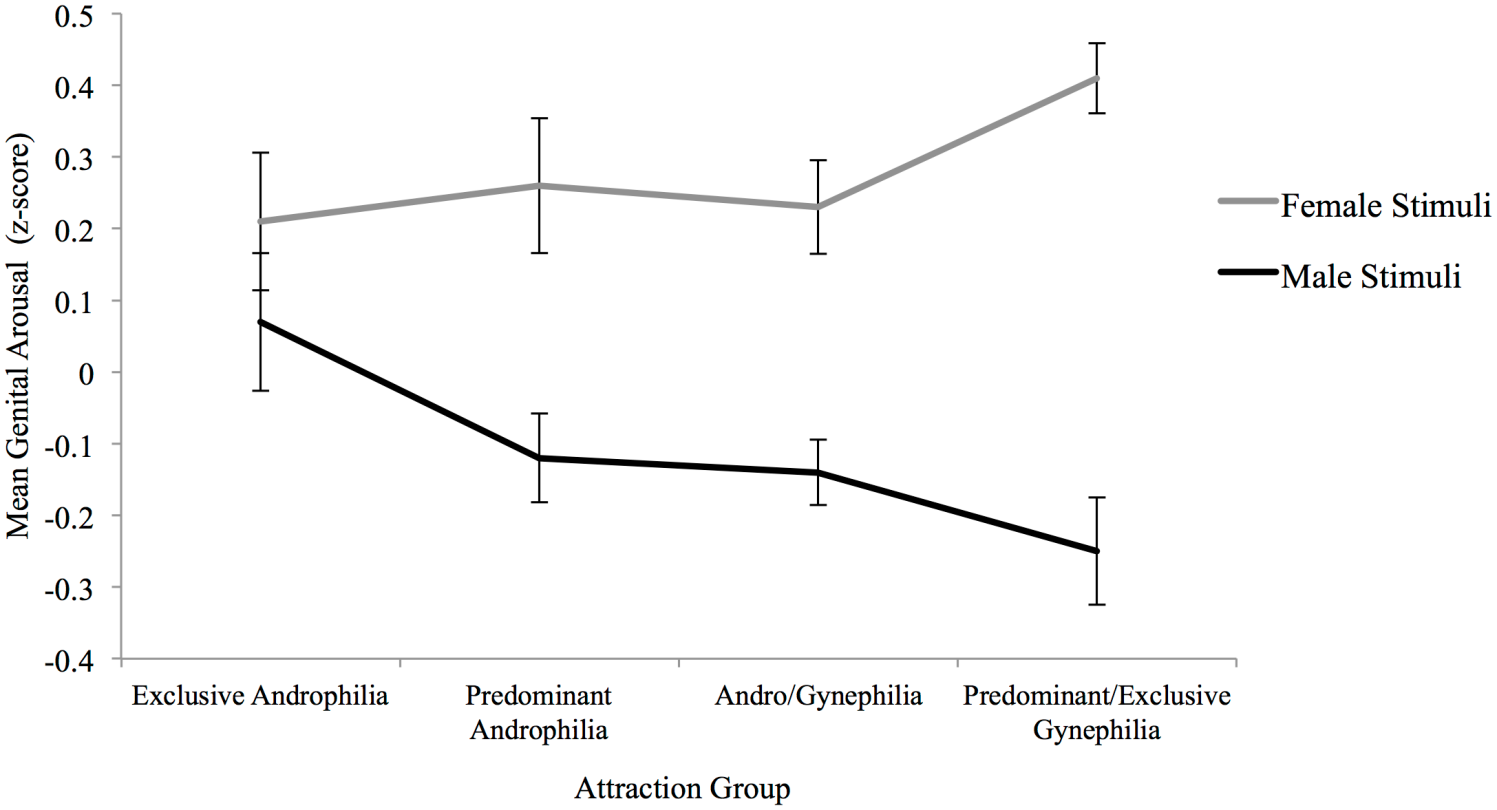
Genital response to female and male audiovisual stimuli by attraction group for Study 1. Exclusive Androphilia (*n* = 14), Predominant Androphilia (*n* = 19), Andro/Gynephilia (*n* = 21), Predominant/Exclusive Gynephilia (*n* = 22). Error bars indicate standard error of the mean.

Mean genital responses were submitted to a 2 (Stimulus Gender) X 4 (Attraction Group) mixed-model ANOVA and a significant interaction between Attraction Group and Stimulus Gender was detected, Wilks’ *Λ* = .897, *F*(3, 72) = 2.74, *p* < .05, *η*_*p*_^*2*^ = .103. Mixed-model *t* tests showed that predominantly androphilic, andro/gynephilic women, and predominantly/exclusively gynephilic women had significantly greater genital response to female than to male stimuli, whereas exclusively androphilic women showed gender-nonspecific genital response (Fig 2). Effect sizes increased with same-gender attraction (Table 3). Bivariate linear regressions revealed that Kinsey sexual attraction ratings significantly predicted genital response to male sexual stimuli, *B* = -.05, *t*(74) = -2.98, *p* = .004, but not female sexual stimuli, *B* = .032, *t*(74) = 1.73, *p* = .09; increasing degree of gynephilic attractions was associated with significantly lower genital responses to male stimuli. When we limited analyses to women who reported a heterosexual sexual identity only, an identical pattern emerged where exclusively androphilic women showed gender-nonspecific responses, and predominantly androphilic and andro/gynephilic women showed greater arousal to female sexual stimuli.

**Table 3:** Genital responses to gender cues by attraction group for Study 1.

Attraction Group	*n*	*t*	*p*	*d*
Exclusive Androphilia	14	1.15	.23	.39
Predominant Androphilia	19	3.79	< .001	1.09
Andro/Gynephilia	21	3.79	< .001	1.43
Predominant/Exclusive Gynephilia	22	7.00	< .001	2.23

## Study 2

### Introduction

Studies investigating gender-specificity of women’s sexual response have typically used audiovisual stimuli depicting gay, lesbian, and heterosexual couples engaging in oral and penetrative sex (e.g., [1, 2, 7]). The intensity of the sexual interactions depicted in these audiovisual representations of coupled intercourse may obscure gender-specific responses, whereas less intense depictions of nude exercise and masturbation can reveal gender-specific responses, particularly among gynephilic women [4]. Similar results have been reported using eye-tracking measures, where lower intensity sexual stimuli (e.g., solitary nude images) evoke gender-specific patterns of early visual attention (e.g., [47]) and visual fixation on nude genital regions [12] among heterosexual women. Also, depictions of gay men having sex may not be the most valid stimuli to assess sexual interest in men for heterosexual women, particularly given evidence that these stimuli can evoke significant negative affect [7].

Chivers and Timmers [5] used audio narratives describing sexual and nonsexual interactions with partners of both genders in varying relationship contexts (stranger, friend, long-term relationship partner) to examine gender-specific sexual responses among heterosexual women. The narratives described low intensity sexual behaviours: interactions culminated in manual sexual touching of the described sexual partner without orgasm, but did not describe oral or penetrative sex or the sexual response of the research participant. Despite the less intense sexual stimuli, heterosexual women experienced similar genital response to male and female stimuli. Conversely, women’s subjective response was gender-specific; women reported significantly greater subjective sexual arousal to male than to female stimuli. In Chivers and Timmers [5], as with other studies of women’s gender-specific sexual responding, groups of heterosexual women were comprised of exclusively and predominantly androphilic women, therefore we wished to examine whether women reporting exclusive androphilic attractions showed different arousal patterns than women with varying degrees of andro- and gynephilia, as in Study 1.

### Materials and Methods

Ethics approval for this research was granted by the Health Science Research Ethics Board at Queen’s University, and the University of Toronto Health Science Network Research Ethics Board. Participants gave written informed consent after orientation to the laboratory setting, study apparati, and study procedures, and after any questions were addressed.

#### Participants

Thirty cisgender women were recruited through advertisements posted on a university campus using eligibility criteria identical to Study 1, and data were pooled with those of Chivers and Timmers [5] for a total *N* of 78; demographic information is based on 76 women, corresponding with usable subjective sexual arousal data. For the total sample, women ranged in age from 18 to 38 years, with a mean age of 21.54 years old (*SD* = 3.77). Fifty percent of the women were in dating relationships, 44.7% were single, 4.0% were engaged, married, or common law, and 1.3% were divorced. The majority of the sample (59.2%) stated that they were of European descent, 26.3% of women identified as Asian, 2.6% as African, 1.3% as Hispanic, 1.3% as Middle Eastern, 2.6% as having mixed-ethnic backgrounds, and the final 6.6% of women identified with other ethnic groups. The majority of the sample had attended or completed post-secondary education; participants were completing or had completed a bachelor’s degree (76.3%), a graduate or professional degree (13.2%), or community college (6.6%). The remaining participants had graduated from high school or equivalent (2.6%) or did not report their level of education (1.3%). All participants received $25 for their time and expenses.

*Sexual Attractions and Sexual Identity*. Participants reported their relative sexual attraction to women and men using a variation of the Kinsey Sexual Attraction Scale [40] and were grouped based on degree of andro/gynephilic sexual attractions: predominantly androphilic (Kinsey 1; *n* = 2), andro/gynephilia (Kinsey 2–4; *n* = 19), and predominantly/exclusively gynephilic (Kinsey 5–6; *n* = 8). Participants from the Chivers and Timmers [5] study were comprised of women reporting exclusive androphilia (Kinsey 0; *n* = 23) and predominant androphilia (Kinsey 1; *n* = 17). A small number of women with andro/gynephilia (Kinsey 2–4; *n* = 5) and predominant and exclusive gynephilia (Kinsey 5–6; *n* = 2) were also assessed, but excluded from Chivers and Timmers’ [5] analyses. One participant did not report her Kinsey Sexual Attraction score and was thus excluded from the analyses. For further demographic information, please see Chivers and Timmers [5]. An item assessing sexual identity (e.g., lesbian, bisexual, heterosexual, other) was administered. See Table 4 for a breakdown of sexual identity by sexual attraction groups.

**Table 4:** Sexual identities by attraction group for Study 2.

Attraction Group	Sexual Identity				
	Heterosexual	Bisexual	Lesbian/Gay	Other	Total *n*
Exclusive Androphilia	23	0	0	0	23
Predominant Androphilia	16	2	0	1*	19
Andro/Gynephilia	4	17	0	4**	24
Predominant/Exclusive Gynephilia	0	1	7	2***	10

*queer heterosexual—attracted emotionally and physically to men, no gender roles in the relationship.

** pansexual; pansexual; unlabelled; queer.

*** unidentified; queer.

#### Apparatus, Materials, Procedures

Study 2 apparati, materials, and procedures were identical to Study 1 except for the experimental stimuli. The audio narratives, 18 in total, were those used by Chivers and Timmers [5]: Stories were composed of 170 to 185 words, and averaging about 90 seconds duration when read aloud by a female actor, in a neutral tone of voice. The first five sentences established the physical setting and relationship context of each interaction. The stories described sexual (12 stories—two exemplars for each gender by relationship context combination) and nonsexual interactions (six stories—one exemplar for each gender by relationship context combination) with strangers, friends, or long-term relationship partners of both genders. The order of presentation of the 18 audio stories was randomized for each participant.

#### Data Exclusion and Reduction

Genital data from three women in the Chivers & Timmers [5] sample, as well as genital and subjective data from one woman in the current sample, were excluded because of equipment problems or unclear VPA signals. Genital and subjective data from one woman in the Chivers & Timmers [5] sample were also excluded because she did not report her sexual attractions. The remaining women (*n*_*subjective*_ = 76, *n*_*genital*_ = 73) demonstrated a 0.5 SD or greater increase in their maximum genital response to any sexual stimulus, relative to their response to the neutral story, and were therefore included [1]. Mean genital response and subjective sexual arousal change scores were derived in a manner identical to Study 1. Preliminary analyses prior to collapsing Stimulus Gender across relationship contexts revealed no three-way interactions between Relationship Context, Stimulus Gender, and Attraction Group therefore genital and subjective sexual response to gender cues was assessed by averaging across relationship contexts (stranger, friend, long-term relationship partner) for female and male stimuli. Data analyses were performed using SPSS (SPSS Inc., Chicago, Il, USA; Version 19.0).

### Results

#### Subjective Sexual Arousal

**Fig 3:**
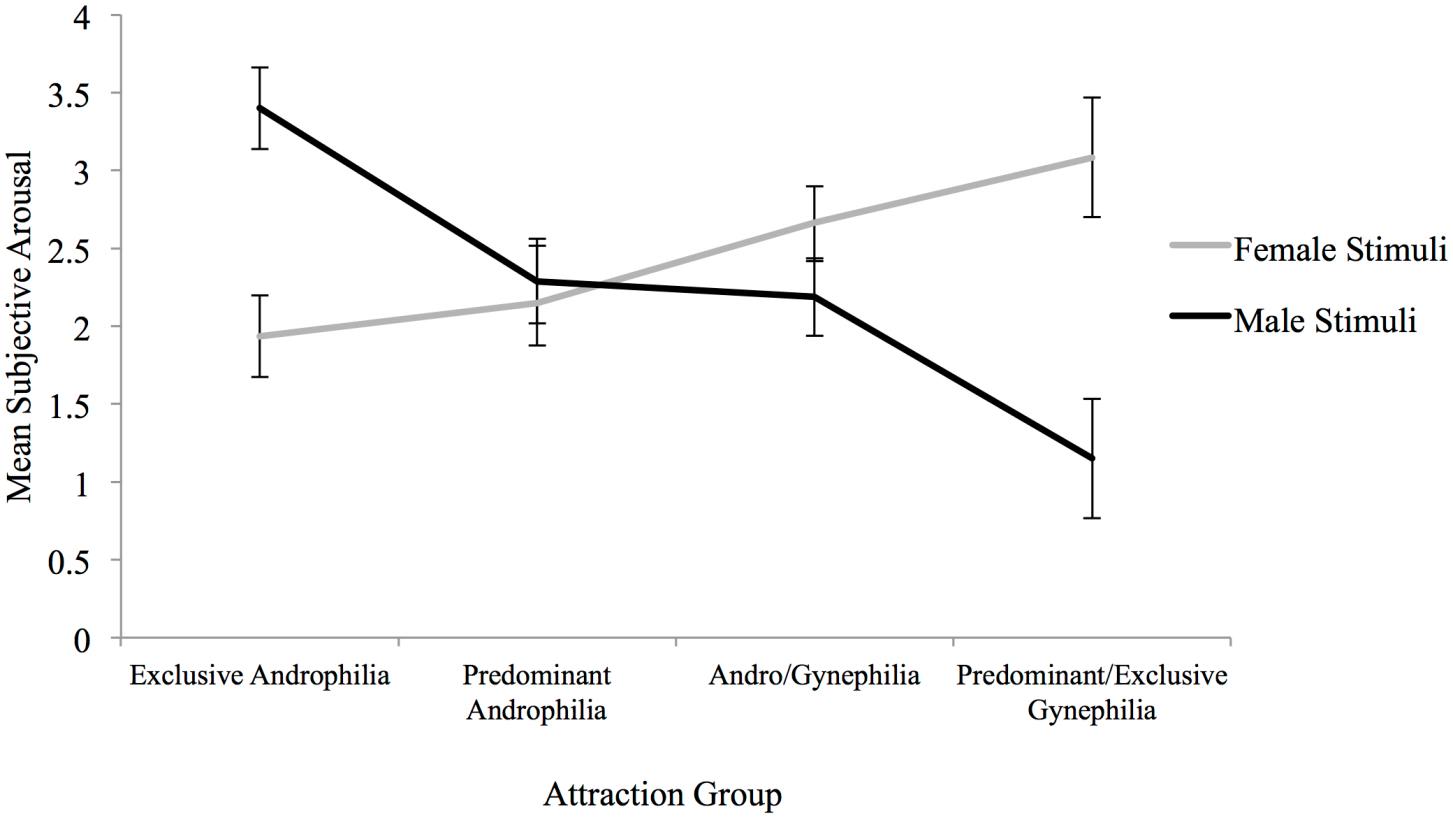
Subjective sexual arousal to female and male narratives by attraction group for Study 2. Exclusive Androphilia (*n* = 23), Predominant Androphilia (*n* = 19), Andro/Gynephilia (*n* = 24), Predominant/Exclusive Gynephilia (*n* = 10). Error bars indicate standard error of the mean.

Mean subjective arousal was submitted to a 2 (Stimulus Gender: female, male) X 4 (Attraction Group: exclusive androphilia, predominant androphilia, andro/gynephilia, predominant/exclusive gynephilia) mixed-model ANOVA revealing a significant interaction between Stimulus Gender and Attraction Group, Wilks’ *Λ* = .546, *F*(3, 72) = 19.99, *p* = < .001, *η*_*p*_^*2*^ = .454. Mixed-model *t* tests showed that exclusively androphilic women reported significantly greater subjective sexual arousal to male than to female stimuli, whereas predominantly/exclusively gynephilic women reported significantly greater subjective sexual arousal to female than to male stimuli (Fig 3). Predominantly androphilic and andro/gynephilic women did not differentiate male and female stimuli in their subjective sexual responses (Table 5). Bivariate linear regressions showed that Kinsey sexual attraction ratings significantly predicted subjective sexual arousal to female sexual stimuli, *B* = .197, *t*(74) = 2.34, *p* < .05, and subjective arousal to male sexual stimuli, *B* = -.40, *t*(74) = -5.16, *p* < .001; gynephilic attractions significantly predicted increases in subjective sexual arousal to female stimuli and decreases to male stimuli.

**Table 5:** Subjective sexual arousal to gender cues by attraction group for Study 2.

Attraction Group	*n*	*t*	*p*	*d*
Exclusive Androphilia	23	-3.91	< .001	1.18
Predominant Androphilia	19	-.34	.73	.10
Andro/Gynephilia	24	1.31	.19	-.41
Predominant/Exclusive Gynephilia	10	3.41	< .001	-1.64

#### Genital Response

**Fig 4:**
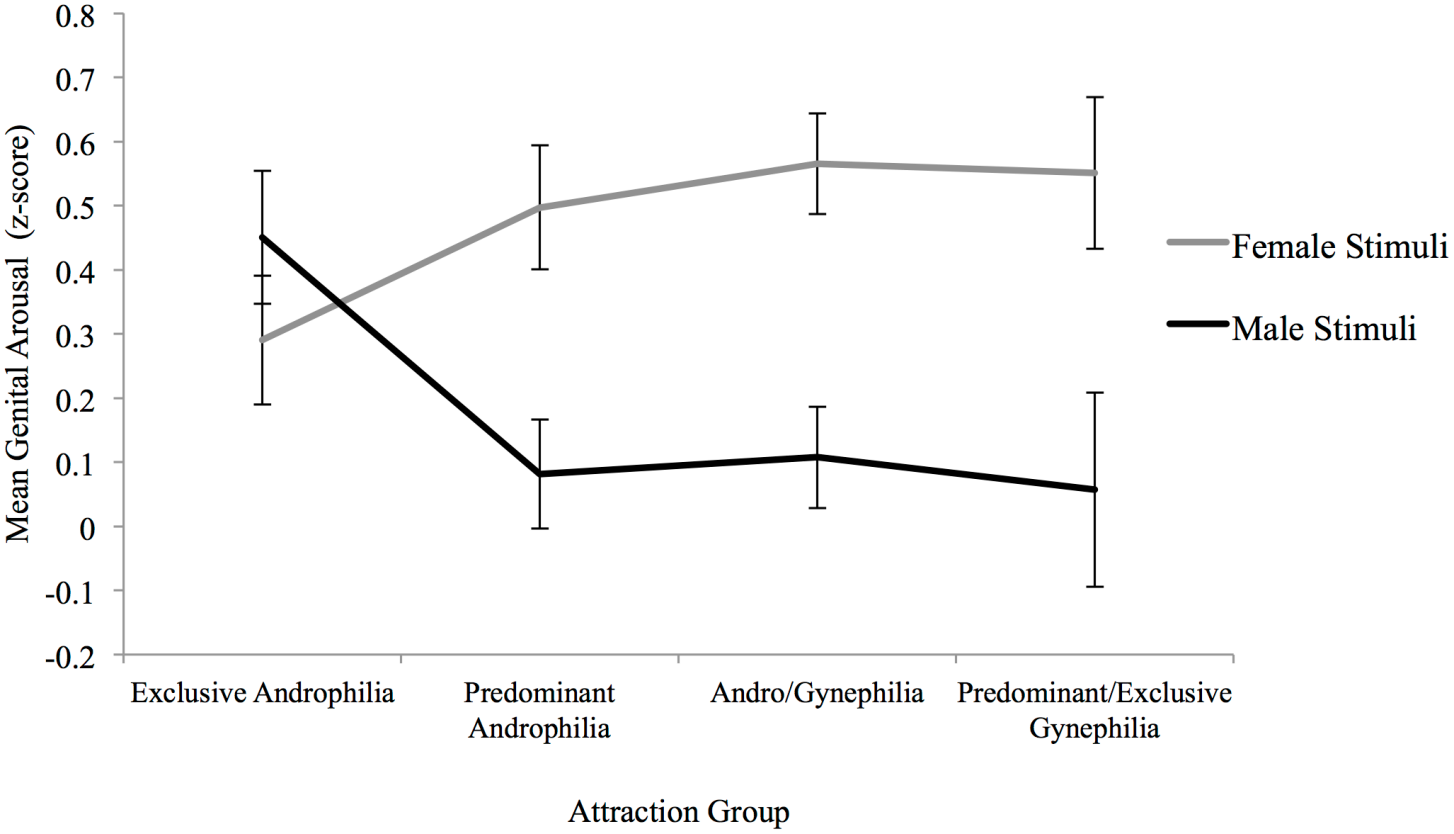
Genital response to female and male narratives by attraction group for Study 2. Exclusive Androphilia (*n* = 20), Predominant Androphilia (*n* = 19), Andro/Gynephilia (*n* = 24), Predominant/Exclusive Gynephilia (*n* = 10). Error bars indicate standard error of the mean.

Mean genital response was submitted to a 2 (Stimulus Gender) X 4 (Attraction Group) mixed-model ANOVA revealing a significant interaction, Wilks’ *Λ* = .882, *F*(3, 69) = 3.08, *p* = .03, *η*_*p*_^*2*^ = .118. Mixed-model *t* tests (Table 2) showed that predominantly androphilic, andro/gynephilic, and predominantly/exclusively gynephilic women had significantly greater genital response to female than to male stimuli, whereas exclusively androphilic women had gender-nonspecific genital responses (Fig 4). Effect size increased with greater gynephilia, except in the two groups reporting highest attraction to women (Table 6). Bivariate linear regressions showed that Kinsey sexual attraction ratings significantly predicted genital response to male sexual stimuli, *B* = -.06, *t*(71) = -2.14, *p* < .05, but not female sexual stimuli, *B* = .036, *t*(71) = 1.31, *p* = .19; increasing degree of gynephilic attractions was associated with lower genital response to male stimuli. When we limited analyses to women who reported a heterosexual sexual identity only, an identical pattern emerged.

**Table 6:** Genital response to gender cues by attraction group for study 2.

Attraction Group	*n*	*t*	*p*	*d*
Exclusive Androphilia	20	-1.22	.23	.36
Predominant Androphilia	19	3.09	< .001	-1.06
Andro/Gynephilia	24	3.82	< .001	-1.20
Predominant/Exclusive Gynephilia	10	2.66	.01	-1.18

## Discussion

Across two studies, only exclusively androphilic women showed a gender-nonspecific pattern of genital response to female and male sexual stimuli. This pattern was demonstrated using both audiovisual and narrative sexual stimuli. All other groups of women demonstrated greater genital responses to the female stimuli. Subjective sexual arousal was more variable; in Study 1, exclusively androphilic women reported gender-nonspecific arousal whereas all other groups reported greater arousal to female stimuli. In Study 2, exclusively androphilic and predominantly/exclusively gynephilic women reported gender-specific sexual arousal; predominantly androphilic and andro/gynephilic women reported similar sexual arousal to female and male sexual stimuli. Across both studies, sexual attraction predicted genital response to male but not female sexual stimuli, with increasing degree of gynephilia associated with lower genital responses to male stimuli. For subjective sexual arousal, sexual attractions significantly predicted both increases in subjective sexual arousal to female sexual stimuli and decreases in subjective arousal to male sexual stimuli.

Although the *a priori* hypothesis that exclusively androphilic women would demonstrate gender-specific sexual responses was not fully supported, we believe an even more intriguing pattern of sexual response was revealed. Three results are particularly noteworthy: First, gender-nonspecific genital response was observed in exclusively androphilic women only, confirming and clarifying previous reports that nonspecific genital responding is characteristic of androphilic women [4]. Second, although predominantly androphilic women showed increased genital responses to both female and male stimuli, their genital responses were significantly greater to female stimuli. In other words, predominantly androphilic women’s sexual arousal was more gender-specific than exclusively androphilic women’s, but not in alignment with their stated sexual attractions and heterosexual sexual identity. Third, and related, women reporting any degree of gynephilia demonstrated greater genital arousal to female stimuli than male stimuli. This pattern is expected for predominantly or exclusively gynephilic women, given the findings of Chivers et al. [4], but this pattern is again not in line with the stated sexual attractions and sexual identities of predominantly androphilic or gyne/androphilic women.

We have speculated that gender-nonspecific sexual responding among women who identify as heterosexual is attributable to heterogeneous sexual attractions among heterosexual samples. As such, we predicted that exclusively androphilic women would show gender-specific patterns of sexual response, whereas predominantly androphilic women would show more variable, gender-nonspecific patterns, thereby resulting in the gender-nonspecific profile typically reported for heterosexual female samples. Indeed, group differences were found but, counter to our prediction, exclusively androphilic women showed gender-nonspecific genital responses, and predominantly androphilic women, along with all other women, showed significantly greater arousal to female sexual stimuli, across two studies. These findings replicate Chivers et al.’s [4] report of gender-nonspecific genital responses only among heterosexual women but are counter to Suschinsky et al.’s [9] results reporting no differences in gender-specific responses between exclusively and predominantly androphilic women.

Subjective sexual arousal followed an identical pattern for Study 1: gender-nonspecific for exclusively androphilic women versus greater arousal to female stimuli for all other women. These results converge with data from other methodologies for assessing sexual interest, demonstrating less differentiated patterns of visual attention to sexual stimuli among androphilic versus gynephilic women (e.g.,[15, 16, 19, 21, 22, 29]). Unlike other research, however, these results suggest greater congruence between genital and subjective sexual responding than is typically reported for women (see Chivers et al. [48]).

In Study 2, exclusively androphilic women reported greater arousal to male than female sexual stimuli, whereas predominantly androphilic women reported arousal that was more similar to andro/gynephilic women. These results are more similar to other studies reporting lower agreement between genital and subjective sexual responses to preferred and nonpreferred sexual stimuli. The differing pattern of results may be related to the greater explicitness and intensity of audiovisual (Study 1) versus audio (Study 2) sexual stimuli. Other possible explanations may include the role of attractiveness in gender-specific patterns of attention to sexual stimuli whereby physical attractiveness cues, more readily observable in visual stimuli, may exert greater influence on self-reported feelings of sexual arousal (see Lippa [21, 22]).

The results for predominantly androphilic women—greater sexual response to female versus male sexual stimuli—further unsettle sexual orientation bound conceptualizations of variability in women’s sexual response (e.g., [49]). Among predominantly androphilic women—who self-identify as heterosexual—genital response was not only significantly different from that of exclusively androphilic women, and significantly gender-specific, but counter to prediction; these women showed significantly greater genital responses to audiovisual and narrative sexual stimuli depicting women. In other words, these women showed a differentiated pattern of genital response that is not aligned with their stated sexual attractions. When we limited our analyses to only those women who identified as heterosexual, thereby excluding 5 women in Study 1 and 3 women in Study 2, the pattern remained the same. Subjective sexual arousal was similarly discrepant, with women reporting about equal arousal to female and male sexual stimuli across the two studies.

Also noteworthy is the replication of this effect across two different modalities—audiovisual and narrative—of sexual stimuli. Previously, it was proposed that nonspecific genital responding in women may be attributable to automatic genital response to visual sexual cues [30], such that gender-nonspecific response likely reflects response to prepotent sexual features ubiquitous to complex sexual stimuli depicting couples or individuals engaging in sexual acts. Gender-nonspecific genital responding does, however, extend to narrative stimuli [5], even narratives describing nonpreferred (among nonmasochistic women) sexual activities such as sexual masochism with female or male dominants [3]. The current data suggest that, for predominantly androphilic women, features common to narrative and audiovisual female sexual stimuli are stronger determinants of genital responding than those present in male sexual stimuli, underscoring the lower relevance of preferred gender cues to sexual response among androphilic women.

One striking aspect of these data is the placement of an empirical fulcrum, a balance point along the continuum of sexual attractions where genital response patterns shifted away from an undifferentiated activation of the sexual response system. Across both studies, patterns of genital response were not dimensionally distributed across women, suggesting possibly meaningful categorical differences in the sexual responses of women who do or do not experience gynephilic attractions. For genital response, the fulcrum was situated at the shift from exclusive androphilia: Women reporting *any* degree of gynephilia, regardless of sexual identity, experienced significantly greater genital response to female than male sexual stimuli. Sexual orientation or directional sexual attractions have been proposed to be taxonic-dimensional, such that some process canalizes development of sexual attractions to females or males or both, although expression of the trait may also vary dimensionally [50–52]. This could suggest a taxon-like quality of sexual attractions and sexual response, independent of sexual identities among women.

Alternatively, sensitivity and arousability to a broader range of sexual stimuli may be related to women’s sexual attractions. Commenting on the proceptive and receptive nature of sexual response, Vrangalova and Savin-Williams [36] proposed that, “…mostly heterosexual individuals may be proceptively oriented toward only one sex, but have higher arousability to a wider range of stimuli, including those of their non-preferred sex” (pp. 97). Gender-nonspecific sexual responding may therefore mean that other cues, such as physical attractiveness (e.g., [21]), relationship context [5] or physical setting [53], are more relevant to activation of the sexual response system than gender cues for androphilic women.

We recently conducted an indirect test of this hypothesis—that gender-specific sexual response is obscured by sexual context in androphilic women—by using prepotent sexual stimuli, that is, images of sexually-aroused genitals as sexual stimuli [54]. Participants viewed slideshows of images of aroused or unaroused male and female genitals with limited to no depicted context (i.e., no other physical features of the sexual targets, no sexual activity, no context provided for the images, and so on). In this study, heterosexual women were those who identified as such and also reported exclusive/predominant androphilia. Both genital and subjective sexual responses were significantly greater to male than female prepotent sexual stimuli, the only published study thus far demonstrating gender-specific genital response in heterosexual women. Gender cues, such as sexually-aroused genitals, are therefore sufficient, but not necessary, cues for genital sexual responses among androphilic women.

Individual differences other than gendered attractions, such as sociosexuality, are meaningfully associated with differentiated patterns of sexual response among women. Sociosexuality is the propensity to engage in sexual activity as a function of relationship investment; individuals high in sociosexuality are more likely to desire, engage in, and have positive attitudes about casual sex. Timmers and Chivers [55] reported that both heterosexual women and men higher in sociosexuality showed greater genital response to stimuli describing sex with unfamiliar than familiar persons, and higher genital response to stimuli depicting low versus high relationship commitment. Among androphilic women, these effects were most pronounced for male sexual stimuli. This could be interpreted as contextual cues interacting with individual differences in sociosexual and gender orientations to influence patterns of sexual response.

Individual differences in sexual activity preferences may also be more relevant to heterosexual women’s sexual response than gender cues. Heterosexual women without sexual interest in masochism showed significantly greater genital and subjective sexual response to narratives describing conventional sex acts versus those describing sexual masochism, and these effects were mostly independent of the gender of actor described in the stimulus [3]. Although “activity-specificity” was demonstrated among exclusively and predominantly androphilic women with conventional sexual interests, these women also showed genital responses to descriptions of masochistic sex and purely masochistic acts such as receiving pain where no overt sexual cues were described; this speaks to the potency of the sexualized context of masochism to evoke sexual response despite the nonpreferred nature of these stimuli for these women.

In light of the current data, the conceptual coherence of a link between women’s stated sexual attractions and patterns of sexual response begins to break down. Resolving the supposed discrepancies between sexual identities, sexual attractions, subjective sexual arousal, and genital responses could be straightforward if we adopted the perspective that our heterosexually-identified women were not truly heterosexual. Greater sexual response to female stimuli among andro/gynephilic women could be indicative that these women are, instead, nascently bisexual or lesbian and we are witnessing their sexuality in an early stage of development, at the dawn of these women’s same-gender sexual careers [56, 57]. Our sample is relatively young and the temporal stability of patterns of sexual response has not yet been established beyond a couple of months [58]. Qualitative data on “mostly heterosexual” women suggests this is a possibility. Diamond’s research on sexual fluidity proposes that sexual minority women’s sexual attractions and identities are subject to an iterative process, whereby women are “continuously undergoing processes of identity exploration, uncertainty, and commitment regarding their same-sex attractions and/or experiences [59].” Longitudinal data on patterns of sexual response and their relation to sexual identities and attractions, in a developmental context, may address this question.

Using sexual psychophysiology data to make pronouncements regarding a woman’s true sexual identity or sexual desires is, however, illogical. The flaws in this reasoning become abundantly clear in the face of data showing that a wide range of nonpreferred sexual stimuli, including nonhuman primates [2, 4] and depictions of sexual coercion [9, 60, 61], are capable of evoking significant genital response in women, in the notable absence of subjective sexual arousal. If we assume that genital response is an objective indicator of a woman’s true sexual attractions and desires, we are left with the frankly absurd conclusion that women are sexually attracted to bonobos or to sexual assault. Sexual identity (how the individual conceptualizes and socially describes their patterns of sexual attractions and desires), sexual attractions (sexual orientation), and sexual response (sexual arousal and desire) are not interchangeable constructs in women, such that a woman’s sexual desires and attractions can be deduced from sexual response patterns (see Chivers [30, 31]).

Instead of using sexual psychophysiology as an arbiter for sexual identities, the current data could provide a window of opportunity to understand factors associated with the incentivization of sexual cues in women; that is, how certain categories or groups of sexual cues obtain their sexual salience, are capable of activating the sexual response system, and inform and direct motivated sexual behaviors. Clarifying the relationship between sexual attractions and sexual responses also has implications for understanding how sexual attractions are incentivized, reinforced, and maintained in women (e.g., Incentive Motivation Model of sexual response [62]), and understanding how sexual orientations emerge and coalesce in women. Exclusively androphilic women demonstrating gender-nonspecific genital responding across two stimulus modalities suggests that cues never once directly associated with sexual reward (e.g., sexual gratification with a female sexual partner) still have the capacity to evoke significant sexual response. Toates [62] proposed that sexual stimuli become incentivized through positive experiences and that the motivational value of incentives is amplified if accompanied by sexual arousal. Positive experiences such as genital vasocongestion and accompanying pleasurable sensations, particularly in the absence of negative affect to female sexual stimuli [5], could therefore incentivize nonpreferred sexual stimuli among androphilic women [63]. Multiple opportunities exist for further incentivization of same-gender sexuality beyond direct sexual contact with women, such as sexual fantasy with or without sexual activity (solitary or partnered) and consumption of sexual media. This potential for flexibility of androphilic women’s sexual expression is evident in studies reporting significant variation in the gender of sexual partner featured in sexual fantasies [64].

Other avenues for future research might include examining learning history and sexual incentivization. Understanding how consumption of sexualized media, where sexual interactions between two women are now commonplace [56], affects sexual response and the range of sexual cues capable of activating the sexual response system, would not only address the puzzle presented by the current data, but would also help to fill in a notable gap in contemporary models of sexual response that consistently leave undefined what features comprise a sexually competent stimulus, that is, a stimulus capable of evoking sexual response [62, 65]. And given the role that sexual arousal is now believed to play in women’s sexual motivation—that the state of sexual desire is activated by the processing of sexually-competent cues and emerges from sexual arousal [62]—elucidating the triggers for sexual arousal and desire may inform treatment of highly prevalent concerns regarding low sexual desire among women [66].

Another approach to understanding gender-nonspecific sexual response in androphilic women is to consider that the motivation to look at and engage with nonpreferred sexual stimuli may not be sexual for some. According the Information Processing Model (IPM) of sexual response [67], competent sexual stimuli with sexual meaning automatically activate physical and psychological sexual responses and reciprocally recruit attention to these stimuli. The Preparation Hypothesis [61] proposes that stimuli with sexual meaning, whether preferred or not, initiate an automatic and protective genital response in women. Sexual stimuli that recruit and maintain attention, for whatever reason, could in theory generate a detectable sexual response. Preliminary data support exploring this possibility, with women’s gender-nonspecific visual attention related to the gender-specificity of sexual response [68]. Exclusively androphilic women may be initially motivated to attend to and scrutinize female sexual stimuli for reasons other than sexual attraction, such as social comparison [11] or intrasexual competition [69–71] however the sexual cues present in these stimuli would be sufficient to generate a physiological sexual response, the result being gender-nonspecific genital vasocongestion. Although a viable and testable explanation for nonspecific sexual responding to visual sexual stimuli featuring attractive women, this would not explain gender-nonspecific genital response to narrative stimuli, as demonstrated in Study 2.

The generalizability of these findings is among the limitations of the current research. Volunteer bias is a perennial concern in sexuality and sexual psychophysiology research, with volunteers reporting more sexual liberal attitudes [72, 73], more sexual partners, more noncoital sexual experiences (oral sex, masturbation) less sexual inhibition, and more interest in and experience with sexually explicit materials [73–75]. With regard to nonspecific sexual responding among heterosexual women, Chivers et al. [1] found nonspecific patterns of genital among less sexually-experienced heterosexual women recruited to reduce volunteer bias.

Another possible concern is the use of vaginal photoplethysmography to assess genital responses in women because this methodology has been criticized by some as a less valid measure of sexual response [76], despite ample demonstration of its construct validity [9, 42, 48]. A number of relevant studies mitigate these concerns because patterns similar to those demonstrated in the current studies have been shown using other sexual psychophysiological methods in a multitude of research settings; these include thermal imaging of genital response [6], viewing time [15, 16, 19, 21, 22, 29], and pupil dilation [18, 77]. Last, gender-specific sexual responding has been shown in a mostly exclusively androphilic female sample using decontextualized sexual stimuli [54], therefore it is not the case that vaginal photoplethysmography produces uninterpretable data. Rather, we believe there is a more intriguing relationship between stimulus features and sexual attractions that, in sexual-context laden sexual stimuli, evokes very different sexual response patterns.

A final concern regarding our findings is whether the differences in specificity of genital responses observed between exclusively versus predominantly androphilic women would replicate with other methods of assessing sexual response and sexual interests. Here an important theoretical and methodological point is raised, where researchers keen to understand correlates of same- and other-sex attractions among women are cautioned to reconsider how sexual attractions are conceptualized and assessed. Future research examining correlates of women’s sexual orientation must consider that incidental same-sex attractions among heterosexual women may not be so negligible and that combining groups of exclusively and predominantly androphilic women is inadvisable. At present, the most parsimonious conclusion that can be drawn is that heterosexually identified women are not a homogenous group with respect to sexual response.
